# Application of a framework for determining number of drugs

**DOI:** 10.1186/s13104-016-2076-5

**Published:** 2016-05-13

**Authors:** Amber M. Goedken, Brian C. Lund, Elizabeth A. Cook, Mary C. Schroeder, John M. Brooks

**Affiliations:** Division of Health Services Research, University of Iowa College of Pharmacy, 115 S. Grand Ave, S557 PHAR, Iowa City, IA 52242 USA; Center for Comprehensive Access and Delivery Research and Evaluation, Iowa City Veterans Affairs Health Care System, Iowa City, IA USA; Department of Epidemiology, College of Public Health, University of Iowa, Iowa City, IA USA; Clinical Trials Statistical and Data Management Center, University of Iowa, Iowa City, IA USA; Arnold School of Public Health, University of South Carolina, Columbia, SC USA

**Keywords:** Drug count, Framework, Pharmacy refill database, Medicare, Acute myocardial infarction

## Abstract

**Background:**

There are many different methodologies used in the literature for determining the number of drugs used by a patient, and many are incompletely described. This may be attributable to the lack of a framework to help investigators choose and describe their methods and the lack of evidence on the implications of the choice. The purpose of the study was to propose a framework and illustrate how that framework can be used to create and succinctly describe various approaches to counting the number of drugs used by patients and to examine the impact of varying individual components of the framework on the resulting drug count.

**Methods:**

The three component framework requires specification of scope, uniqueness, and timeframe. The framework was applied to Medicare beneficiaries admitted for acute myocardial infarction in 2008. Drug use was ascertained by Part D prescription drug event files. A default measure for drug count was established, and fourteen additional measures were created by separately altering individual components of the default to illustrate the application of the framework and understand how these changes impacted drug count. Median drug counts and the frequency distributions of beneficiaries experiencing a change in count from default were produced for each measure.

**Results:**

The median drug count for the default measure was 4. Alteration of the timeframe component had the largest impact on drug counts, with a look-back period of 180 days producing a median count of 8 and changing the count by at least two for 73 % of patients. Variations of the other components had less impact.

**Conclusion:**

Our framework is intended to be used by investigators to select an approach to counting number of drugs in their studies. Extending the timeframe over which fills from a pharmacy refill database could be counted toward the drug count produced the greatest changes in the number of drugs.

## Background

Determining the number of drugs used by a patient is necessary for a variety of research applications. For example, an investigator’s objective may be to assess trends in drug use within a population [1] or seek predictors of the number of drugs used [2]. Number of drugs may serve as an indicator of comorbidity [3] or regimen complexity [4] and be included as a covariate. Regardless of purpose, an investigator must select a method for measuring number of drugs and is confronted with a plethora of approaches that have been used in the existing literature. Often these approaches are incompletely described, if at all, and therefore difficult to replicate. One possible contributor to the abundance of methodologies and lack of adequate description is the absence of a framework to assist investigators in selecting and succinctly describing their methods.

Another possible contributor to the variety of approaches used is the lack of studies demonstrating the impact of the chosen methodology on patient drug counts. The few available studies have provided descriptive statistics regarding the distribution of number of drugs under selected methods [5–7] but have not characterized how drug counts are sensitive to altering specific components of a measure. Differences in counts between two studies using non-uniform methods can lead to misleading inferences. For example, the effect of drug counts on adherence to a newly prescribed drug may vary across two studies. These inconsistent results may simply be caused by different methods to count drugs across studies. Evidence on how many patients’ counts change and how much their counts change with particular changes in methodology is needed to aid in the evaluation of the robustness of published study findings examining number of drugs.

Therefore, the first objective of this study was to propose a framework and illustrate how that framework can be used to create and succinctly describe various approaches to counting the number of drugs used by patients. The second objective was to examine the impact of varying individual components of the framework on the resulting drug count, using a pharmacy refill database as the source of drug regimen information, and applied to a cohort of Medicare patients with Part D benefits that were admitted to an acute hospital for acute myocardial infarction (AMI).

## Methods

### Framework

We propose a three component framework to be considered when creating and describing methods for counting the number of drugs used by a patient (Table 1). Importantly, we do not advocate the creation of a single standard measure, as it should be allowed to vary according to study specific objectives. This framework applies regardless of the purpose for measuring number of drugs and requires the specification of three components. The first component is scope, which refers to the types of products that will be included or excluded as being drugs. Examples of products that could be excluded are over-the-counter drugs or drugs with specific routes of administration. The second component is uniqueness, which describes how drugs should be counted where combination products with multiple active ingredients are concerned and in the case where a single ingredient may be found in multiple products in a regimen. The basic approaches are to count unique ingredients or unique products. The final component is timeframe, which requires specification of a cross-sectional versus longitudinal orientation. Each component requires the investigator to establish a conceptual definition and then select operational criteria that best approximate the conceptual definition as allowed by the available data. As an example related to the timeframe component, a commonly used operational criterion is the number of drugs dispensed in the 6 months or year prior to an index event. The intended conceptual definition may be a longitudinal orientation.

**Table 1:** Three component framework for constructing conceptual definitions and operational criteria for counting number of drugs

Component	Description and explanation/examples
1. Scope	What types of products will be considered drugs? Each drug product used by a patient can be classified according to these five subcomponents: (a) prescription drug status, (b) drug type, (c) route of administration, (d) dosage form, and (e) common use. This is not an exhaustive list of subcomponents. The investigator can create additional subcomponents as needed for the specific study objectives, such as therapeutic category or pregnancy category. Possible values for each of the five subcomponents are given below for illustrative purposes and are not intended to be exhaustive Prescription drug status: prescription drug, over-the-counter drug Drug type: standard drug, vitamin, herbal, dietary supplement, other complementary and alternative medicine Route of administration: oral, inhalation, topical, transdermal, ophthalmic, subcutaneous, sublingual, rectal Dosage form: tablet, capsule, cream, solution, suspension Common use: regular, as needed For each subcomponent, decisions must be made about which values will be counted as drugs, even if the decision is to include all possible values for a subcomponent. For example, will prescription and over-the-counter products be counted as drugs? Will vitamins, herbals, dietary supplements, or other various complementary and alternative medicine products be counted in addition to standard drugs? Will certain routes of administration be excluded? Will all dosage forms be counted? Will drug products commonly used on an “as needed” basis be included along with those commonly used on a regular basis?
2. Uniqueness	Will an ingredient-based or product-based approach be used? In an ingredient-based approach, drug products are dissected into their ingredients, and unique ingredients are counted. Single ingredient products contribute one to the count while combination products contribute at least two to the count of ingredients. When multiple drug products include the same ingredient, that ingredient is counted only once. When drug products are dissected into their ingredients, those ingredients can be counted based solely on ingredient (e.g. albuterol), or descriptive information can be appended to an ingredient to count at a more detailed level of ingredient-route (e.g. albuterol-oral), ingredient-route-form (e.g. albuterol-oral-tablet), or ingredient-route-form-strength (e.g. albuterol-oral-tablet-2 mg). For the sake of illustration, consider a regimen including: (1) a combination inhaler containing albuterol and ipratropium, (2) albuterol nebulizer solution, and (3) albuterol oral tablets. The ingredient-based count at the ingredient level is 2 drugs, 1 for albuterol and 1 for ipratropium. The ingredient-based count at the ingredient-route level is 3 drugs, 1 for albuterol-inhalation, 1 for ipratropium-inhalation, and 1 for albuterol-oral In a product-based approach, unique drug products are counted. Combination products contribute one to the count regardless of the number of ingredients in the product. Drug products can be counted at the ingredient(s)-route-form-strength level (e.g. albuterol/ipratropium-inhalation-aerosol-100 mcg/20 mcg) or at a more general level of ingredient(s)-route-form (e.g. albuterol/ipratropium-inhalation-aerosol), ingredient(s)-route (e.g. albuterol/ipratropium-inhalation), or ingredient(s) (e.g. albuterol/ipratropium). Considering the illustrative regimen described above, the product-based count at the ingredient(s)-route-form level is 3 drugs, 1 for albuterol/ipratropium-inhalation-aerosol, 1 for albuterol-inhalation-solution, and 1 for albuterol-oral-tablet
3. Timeframe	Will a cross-sectional or longitudinal orientation be used? For cross-sectional analyses, what criteria will be used to determine whether a drug was in the regimen at the specified point in time? Cross-sectional vs. longitudinal orientation. A cross-sectional orientation seeks to count the number of drugs in a regimen at a specific point in time. In contrast, the longitudinal orientation asks how many unique drugs the patient has used over some period of time (e.g. 1 year). Thus, the longitudinal orientation includes all drugs present in the regimen at the end of the time interval plus all drugs that had been used and discontinued during the interval Timeframe criteria for cross-sectional analyses. The choice of criteria for the cross-sectional orientation is driven by the data source. Drug use data collected from patient interview or questionnaires will often ask the patient about what drugs they took yesterday or within some relatively short time frame (e.g. last 7 days). Studies using inpatient drug administration records would typically count unique drugs administered on a given day. Pharmacy refill databases are another common data source and require a more thorough examination (Fig. 1)
Other considerations	Additional considerations can be implemented using variations of these 3 basic components to accomplish study specific objectives. Some examples using a standard specification for most drugs, but applying different criteria for selected types of drugs based on study specific needs are: A study focused on asthma may apply specialized criteria for inhaled dosage forms A study focused on pain management may apply a wider allowable index gap (defined as the length of time before the index date in which a drug must be filled to be counted, Fig. 1) for drug products commonly used on an “as needed” basis

### Patients and data source

Patients examined in this analysis were drawn from a study comparing the effectiveness of AMI combination treatments on survival, side effects, and costs [8]. Medicare enrollment and medical claim files from the Chronic Condition Data Warehouse data were used to select patients. Beneficiaries were required to be admitted and discharged for an AMI in 2008, with no history of AMI in the prior 12 months. Their index date was the date of admission. They were also required to survive for at least 30 days following discharge from their index hospitalization and not have any inpatient, skilled nursing facility claim, or hospice claims during this period. All beneficiaries had continuous fee-for-service Medicare Part A, B, and D coverage in the 12 months before the index hospitalization and until death or 12 months after hospitalization, whichever came first. Additional selection criteria included residence in the lower 48 US states and age ≥66 years old at index date.

### Drug use data

Drug use by AMI patients during 2007 through 2009 for drug counts was ascertained by Part D prescription drug event (PDE) files from the Chronic Condition Data Warehouse. Specific product information (e.g. ingredients, route, dosage form, etc.) was identified by linking these files to a commercially available drug information resource (Multum Lexicon) using National Drug Codes (NDC) (http://www.multum.com/lexicon.html). PDEs for medical supplies were excluded. PDEs that could not be matched or contained days supplied values of 0 or 999 were excluded as needed to apply the various criteria for determining drug count, resulting in a loss of less than 0.05 % of PDEs.

### Variant measures for drug count

**Fig. 1:**
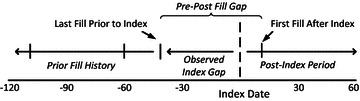
Three key elements in using pharmacy refill databases to determine a cross-sectional drug count. (1) *Allowable index gap*: The first element is the allowable index gap, which is the length of time before the index date in which a drug must be filled to be counted. The investigator assigns a fixed or flexible allowable index gap. A fixed allowable index gap does not vary by fill (e.g. 90 days for all fills). A flexible allowable index gap varies between fills because it is based on data specific to a fill (e.g. days supply of the last fill prior to index). The observed index gap is the time between the last fill prior to index and the index date. If the observed index gap is less than the allowable index gap, then the drug is included in the count. (2) *Post-index fills:* The second element is whether to consider fills that occur after the index date. This involves calculating the observed pre-post fill gap, which is the time between the last fill prior to index and the first fill after index. If the observed pre-post fill gap is less than the fixed (e.g. 90 days) or flexible (2 times the days supply of the last fill prior to index) allowable pre-post fill gap assigned by the investigator, then the drug is included in the count. (3) *Prior fill history:* The third element is whether to consider fills that occurred further back in time than the last fill prior to index. The rationale is to estimate the drug supply the patient had on-hand at the time of the last prior fill. For example, if the patient’s last fill prior to index occurred 27 days following a prior fill of 30 days supply, they would be estimated to have 3 remaining days on hand, which can be incorporated into the allowable index gap for this drug

A default measure for drug count was first established in order to examine the impact of varying individual components of the three component framework. For the default, scope was limited to prescription drug products, uniqueness was determined using a product-based approach, and timeframe was cross-sectional (Table 2). Additional measures were created by separately altering individual components to understand how these changes impacted drug count, leaving the remaining two components unaltered and as specified for the default. A total of 14 variations were created, for illustration, and should not be perceived as all possible variations that could be appropriate for particular research applications (Table 2). Counts under each of the 15 measures were generated for each AMI patient. To illustrate the application of each drug count measure, drug counts for a hypothetical patient (Table 2) were calculated from a hypothetical fill history (Table 3). Variants 1–4 are alterations of scope. Variant 1 altered the values allowed under the prescription drug status subcomponent. Variants 2 and 3 altered the values allowed under the route of administration subcomponent. Variant 4 altered the values allowed under the common use subcomponent. Variants 5–6 are alterations of uniqueness. Both variants changed to an ingredient-based approach but variant 5 was operationalized at the ingredient level and variant 6 was operationalized at the ingredient-route level. Variants 7–14 are alterations of timeframe but all maintain a cross-sectional orientation. Variants 7–9 altered the allowable index gap, defined as the length of time before the index date in which a drug must be filled to be counted (Fig. 1). Variant 10 incorporated post-index fills, variant 11 incorporated the prior fill history, variant 12 incorporated hospitalizations, and variants 13–14 apply different allowable index gaps to different drugs. The allowable index gap and other elements of Fig. 1 are only applicable to the timeframe component and only when using pharmacy refill databases.

**Table 2:** Description of drug count measures and impact of single component variations on drug count

Variant # and label	Scope^a^	Uniqueness^a^	Timeframe^a^	Drug counts for hypothetical patient^b^	Drug count percentiles: 25th, 50th, 75th, 95th	Proportion (%) of beneficiaries with a change in drug count from default: no change, 1 drug, ≥2 drugs
Default	*Conceptual definition*: Prescription drug products *Operational criteria*: Included products flagged as being prescription drugs. All values for remaining subcomponents were included	*Conceptual definition*: Product-based approach *Operational criteria*: Counted unique drug products at the ingredient(s)-route-form level by counting a combination product once, regardless of the number of ingredients in the product	*Conceptual definition*: cross-sectional: the day of hospital admission for AMI, labeled as the index date *Operational criteria*: counted drugs where the observed index gap was less than a flexible allowable index gap of the days supply	3	2, 4, 6, 11	–
1. Rx and OTC products	*Conceptual definition*: Prescription and over-the-counter drug products *Operational criteria*: Included products regardless of whether they were flagged as being prescription or over-the-counter drugs			3	2,4, 6, 11	97, 3, <1
2. Oral Rx products	*Conceptual definition*: Orally administered prescription drug products *Operational criteria*: Included products flagged as prescription drugs and labeled with an oral route of administration			2	2, 4, 6, 10	74, 18, 9
3. Non-topical Rx products	*Conceptual definition*: Prescription drug products administered via any route except for topical application *Operational criteria*: Included products flagged as prescription drugs and labeled with a non-topical route of administration			3	2, 4, 6, 11	96, 3, <1
4. Regularly used Rx products	*Conceptual definition*: Prescription drug products that are commonly used on a regular basis *Operational criteria*: Included products flagged as prescription drugs but excluded products identified by Lund and colleagues as commonly used “as needed” [10]			3	2, 4, 6, 10	80, 17, 4
5. Ingredient-based at ingredient level		*Conceptual definition*: Ingredient-based approach *Operational criteria*: Dissected drug products into ingredients and counted unique ingredients at the ingredient level by counting the same ingredient once, regardless of route or how many products contained the ingredient		4	2, 4, 7, 11	70, 24, 5
6. Ingredient-based at ingredient-route level		*Conceptual definition*: Ingredient-based approach *Operational criteria*: Dissected drug products into ingredients and counted unique ingredients at the ingredient-route level by counting the same ingredient-route pair once, regardless of dosage form or how many products contained the ingredient-route pair		4	2, 4, 7, 11	71, 24, 5
7. Index gap: DS * 1.2			*Conceptual definition*: cross-sectional: the day of hospital admission for AMI, labeled as the index date *Operational criteria*: counted drugs where the observed index gap was less than a flexible allowable index gap of 1.2 times the days supply	3	2, 5, 7, 11	71, 18, 11
8. Index gap: fixed, 90 days			*Conceptual definition*: cross-sectional: the day of hospital admission for AMI, labeled as the index date *Operational criteria*: counted drugs where the observed index gap was less than a fixed allowable index gap of 90 days	4	3, 6, 9, 15	27, 20, 53
9. Index gap: fixed, 180 days			*Conceptual definition*: cross-sectional: the day of hospital admission for AMI, labeled as the index date *Operational criteria*: counted drugs where the observed index gap was less than a fixed allowable index gap of 180 days	4	5, 8, 12, 19	14, 13, 73
10. Post-index, first fill: pre-post fill gap: DS * 2			*Conceptual definition*: cross-sectional: the day of hospital admission for AMI, labeled as the index date *Operational criteria*: considered fills after the index date. Counted drugs where the observed index gap was less than a flexible allowable index gap of the days supply. Also counted drugs that did not meet this criterion but met the following criterion: the observed pre-post fill gap was less than the flexible allowable pre-post fill gap of 2 times the days supply	3	2, 4, 7, 11	75, 17, 9
11. Prior fill history: 180 day cabinet supply			*Conceptual definition*: cross-sectional: the day of hospital admission for AMI, labeled as the index date *Operational criteria*: considered the prior fill history. Counted drugs where the observed index gap was less than a flexible allowable index gap of the days supply plus the drug supply on-hand accumulated from all the fills 180 days before the last fill prior to index	3	2, 4, 7, 11	88, 9, 3
12. Other: hospitalization adjustment: during index gap			*Conceptual definition*: cross-sectional: the day of hospital admission for AMI, labeled as the index date *Operational criteria*: adjusted for hospitalization that occurred during the observed index gap. The rationale is that during hospitalization, the home drug supply is not used because the beneficiary’s drugs are provided by the hospital, and this should be accounted for when determining the allowable index gap. Counted drugs where the observed index gap was less than a flexible allowable index gap of the days supply plus the number of inpatient days during the observed index gap	3	2, 4, 7, 11	93, 4, 3
13. Other: PRN adjustment: fixed index gap, 180 days			*Conceptual definition*: cross-sectional: the day of hospital admission for AMI, labeled as the index date *Operational criteria*: counted drugs where the observed index gap was less than a flexible allowable index gap of the days supply or, for drug products commonly used on an “as needed” basis, where the observed index gap was less than a fixed allowable index gap of 180 days	4	2, 5, 7, 12	60, 27, 13
14. Other: dosage form adjustment: metered-dose inhalers, fixed index gap, 180 days			*Conceptual definition*: cross-sectional: the day of hospital admission for AMI, labeled as the index date *Operational criteria*: counted drugs where the observed index gap was less than a flexible allowable index gap of the days supply or, for metered-dose inhalers, where the observed index gap was less than a fixed allowable index gap of 180 days	3	2, 4, 7, 11	93, 6, 1

^a^Blank column indicates component unaltered and as specified for the default

^b^Drug counts based on fill history for hypothetical patient (Table 3)

**Table 3:** Fill history for a hypothetical patient obtained from a pharmacy refill database

Fill date	Medication	Quantity	Days supply
04/19/08	Estradiol 0.025 mg/24 h transdermal patch	4	28
04/19/08	Metoprolol succinate 50 mg extended-release tablet	30	30
04/19/08	Ezetimibe/simvastatin 10/10 mg tablet	30	30
04/19/08	Nitroglycerin 0.4 mg sublingual tablet	25	9
05/20/08	Estradiol 0.025 mg/24 h transdermal patch	4	28
05/20/08	Metoprolol succinate 50 mg extended-release tablet	30	30
05/20/08	Ezetimibe/simvastatin 10/10 mg tablet	30	30
06/18/08	Estradiol 0.025 mg/24 h transdermal patch	4	28
06/18/08	Metoprolol succinate 50 mg extended-release tablet	30	30
06/18/08	Ezetimibe/simvastatin 10/10 mg tablet	30	30
07/01/08	Admission to hospital for AMI		
07/03/08	Discharge from hospital		
07/03/08	Lisinopril 10 mg tablet	30	30
07/19/08	Estradiol 0.025 mg/24 h transdermal patch	4	28
07/19/08	Metoprolol succinate 50 mg extended-release tablet	30	30
07/19/08	Ezetimibe/simvastatin 10/10 mg tablet	30	30

### Data analysis

Drug count for each count measure algorithm was expressed by the median and the 25th, 75th, and 95th percentiles. In addition, frequency distributions of the proportion of beneficiaries experiencing a change in count of 0, 1 or ≥2 drugs between the default measure and each variation of the default were produced. Analyses were performed using SAS 9.4. The University of Iowa institutional review board approved the research and waived the requirement for informed consent (IRB ID # 200908724).

## Results

Sixty percent of beneficiaries were more than 75 years old, 43 % were male, and 83 % were non-Hispanic whites. Thirty-four percent were dually enrolled in Medicare and Medicaid at the time of AMI hospitalization, and 6 % received the low-income subsidy. Fourteen percent were in the Medicare Part D “donut hole”. Eighty-two percent had uncomplicated hypertension, 68 % had hyperlipidemia, 37 % had diabetes, 30 % had heart failure, 26 % had COPD, 18 % had chronic kidney disease, and 7 % had asthma.

Using the default measure, the median count of drugs was 4 (Table 2). Most variations did not result in different median or 25th percentile values, but yield differences in the higher tail of the distribution (75th and 95th percentiles). Variations that produced a difference in median included lengthening the allowable index gap to 1.2 times the days supply (variant 7), allowing an allowable index gap of 90 days (variant 8), allowing an allowable index gap of 180 days (variant 9), and expanding the allowable index gap for drugs commonly used on an “as needed” basis (variant 13).

The impact of variations was also examined in terms of the proportion of patients who experienced a given change in drug count compared to the default measure. Drug count values remained unchanged in the vast majority of patients (>90 %) for the variations inclusion of over-the-counter products (variant 1), exclusion of drugs administered by a particular route (e.g. topical, variant 3), adjustment to the allowable index gap for hospitalization (variant 12), and expansion of the allowable index gap for a specific dosage form (e.g. metered-dose inhalers, variant 14) (Table 2). In contrast, fewer than 30 % of drug counts were unaffected when index gaps of 90 days (variant 8) and 180 days (variant 9) were allowed. The majority (>50 %) changed by at least two drugs for variants 8 and 9, and over 10 % changed by one drug.

## Discussion

The objective of this study was to propose a three component framework, demonstrate how operational criteria for number of drugs can be created using the framework, and then examine the impact of altering various components. Looking across variants, the most substantial changes in the number of drugs were seen with alterations to the timeframe component. Compared to the default measure, applying a fixed allowable index gap (i.e. look-back period) of 90 days (variant 8) increased the median drug count from 4 to 6, and counts changed by at least two drugs for 53 % of patients. Extending the look-back period to 180 days (variant 9) increased the median drug count to 8, and counts changed by at least two drugs for 73 % of patients. The drugs counted under variants 8 and 9 but not under the default include drugs to which the patient was nonadherent, drugs taken on an “as needed” or otherwise irregular basis, and drugs taken in the past but no longer in use. Jackevicius and colleagues used a look-back period of 120 days and found a median of 5 drugs for elderly patients admitted for AMI, supporting our results that look-back periods of ≥90 days produce higher medians than the default measure [9]. The changes in patients’ counts between methods highlight the need for future studies examining the consistency of results using different methods to count drugs. Other alterations of the timeframe component had less impact than extending the look-back period.

A limited number of studies have previously examined the impact of altering timeframe criteria on drug count. Bjerrum and colleagues compared the mean number of drugs used each day over one year to fills in a 3-month window during that year [5]. The 75th percentile increased, as it did when we changed the operational criteria for timeframe from the default specification to a 90-day look-back period. However, their 75th percentile increased by less than one drug, from 0.3 to 1, while ours increased by three drugs. Fincke and colleagues compared the mean number of drugs used on 40 days over an 18-month study period to fills in a 178-day window during that study period [6]. The mean number of drugs increased by nearly one drug, from 2.63 to 3.54, but when we changed from the default specification to a 180-day look-back period, our median increased by four drugs. They also compared fills in a 132-day window to fills in the 178-day window. Again, the increase in the mean was less than one drug, from 3.06 to 3.54. When we extended the look-back period from 90 to 180 days, the median drug count increased by 2. Timeframe alterations may have had a greater impact in our study because our population of patients hospitalized for AMI was more severely ill with more comorbid conditions, necessitating more drug use and drug changes over time than their populations without index events. In addition, the shift in 75th percentile values in our study could reflect a growing subgroup of patients taking a very large number of drugs, which skews the upper end of the drug count distribution. We are unable to compare the proportion of patients who experienced a change in drug count with other studies because no studies have characterized how many patients’ counts change or how much their counts change with changes in timeframe criteria.

Alterations to the scope and uniqueness components had little impact on the number of drugs. The large proportion of counts unaltered when restricting to orally administered prescription drugs (variant 2) indicates most regimens were composed of only oral drugs. The large proportion unaltered when expanding to include over-the-counter products (variant 1) means most regimens were composed of only prescription drugs or the majority of over-the-counter drugs used by patients were not counted. Few over-the-counter products appear in the data, as they are generally not reimbursed by Medicare Part D plans. The large proportion unaffected when changing uniqueness from a product-based to ingredient-based approach implies most regimens included only single ingredient products, and most did not contain the same ingredient administered via different routes.

The results are not intended to imply that one operational criterion is more appropriate to use than another. The appropriateness of a particular criterion depends on the study objectives and desired conceptual definition. Thus, conceptual definitions need to be specified so judgment can be made on whether the chosen operational criteria are appropriate for the conceptual definition. For example, variants 8 and 9 are more likely than the default to count drugs that were used in the past but are no longer in use, making the count a summation of drugs taken at a point in time and drugs no longer in use. When a cross-sectional orientation is desired, careful evaluation needs to be made to determine if look-back periods of more than 90 days are appropriate. Assessing when certain operational criteria are inappropriate for a particular conceptual definition was outside the scope of this study, but a future study examining this is warranted.

Several limitations must be considered when interpreting our findings. First, we used a pharmacy refill database as our source of drug regimen information and did not examine other sources such as patient interview or drug administration records. These data sources would be more likely to contain use of over-the-counter drugs, drugs used on an “as needed” basis, and drugs used only in specific circumstances, like antibiotic prophylaxis for dental procedures. While we found little impact of the scope component of our framework when applied to pharmacy refill databases, this component may have a greater impact in other data sources where these drugs are more thoroughly captured. In addition, pharmacy refill databases do not provide information about other products not reimbursed by the plan, including drugs purchased out-of-pocket and drug samples. It is unclear what impact the addition of these drugs would have on our findings. A further limitation is that we generated results for a single clinical population. Other components of the framework may be more important in different clinical populations. For example, drug counts in children hospitalized for an asthma exacerbation would be more impacted by methodology variants to route of administration, where drugs administered by inhaler or nebulizer are ubiquitous. Further research applying our framework to drug count methodology variations in other data sources and clinical populations is clearly needed.

## Conclusion

We contribute to the literature by proposing a framework that can be used by investigators selecting the approach for counting number of drugs for their studies. This framework offers an avenue for succinctly describing the chosen approach, which is critical when brevity is required. For example, the investigator can refer to the three components without describing them at length. Our results highlight the need to carefully select and describe methodology for counting number of drugs. Reporting of the conceptual definition and operational criteria for timeframe is particularly important when the source of drug regimen information is a pharmacy refill database. For a cohort of elderly patients, extending the timeframe over which fills from a pharmacy refill database could be counted toward the drug count on the day of admission for AMI changed the number of drugs by at least two drugs for most of the cohort. Future research using other sources of drug regimen information and additional populations are needed to fully understand how the methodology chosen for counting number of drugs influences the counts.

## Abbreviations

AMI: acute myocardial infarction
NDC: National Drug Code
PDE: prescription drug event
